# COVID-19 on resonance magnetic: an incidental but important finding in times of pandemic

**DOI:** 10.31744/einstein_journal/2020AI5891

**Published:** 2020-11-05

**Authors:** José Vitor Rassi Garcia, Eduardo Kaiser Ururahy Nunes Fonseca, Rodrigo Caruso Chate, Daniel Giunchetti Strabelli, Lucas de Pádua Gomes de Farias, Bruna Melo Coelho Loureiro, Lorena Carneiro Ferreira, Márcio Valente Yamada Sawamura

**Affiliations:** 1 Universidade de São Paulo Faculdade de Medicina Hospital das Clínicas São PauloSP Brazil Hospital das Clínicas, Faculdade de Medicina, Universidade de São Paulo, São Paulo, SP, Brazil.

**Figure 1:**
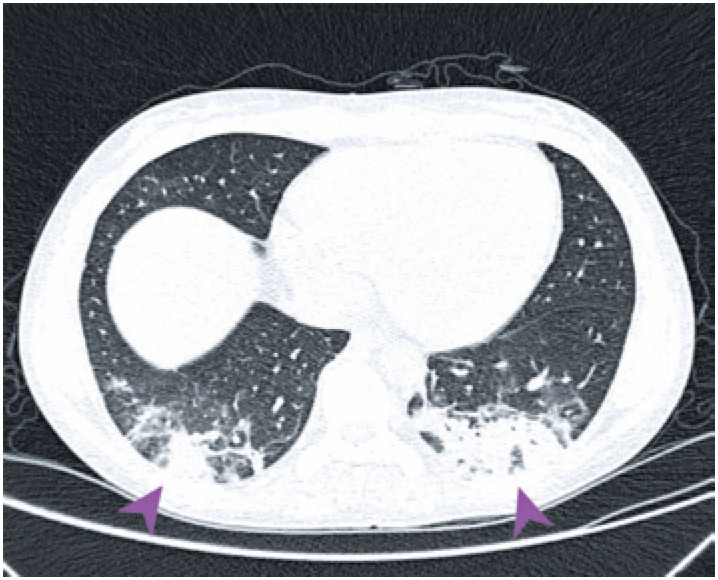
Axial chest computed tomography without contrast showing bilateral ground-glass opacities in lung bases, with peripheral and posterior predominance, suspected of being affected by COVID-19

A 34-year-old woman with a personal history of ulcerative rectocolitis for 5 years who were using sulphasalazine and mesalazine. The patient was admitted to the emergency unit presenting asthenia, dyspnea, fever, cough with hemoptoic sputum for 8 days, and oxygen saturating by 97% in room air. The computed tomography (CT) (Figure 1) showed sparse ground-glass opacities, although more evident in the posterior contour of lower lobes. She was hospitalized and tested positive for coronavirus infection identified through real-time reverse-transcription-polymerase-chain-reaction (RT-PCR) test. During the hospitalization she presented diffuse abdominal pain with liquid, dark, and foul-smelling stools, and underwent an upper abdominal magnetic resonance.

**Figure 2:**
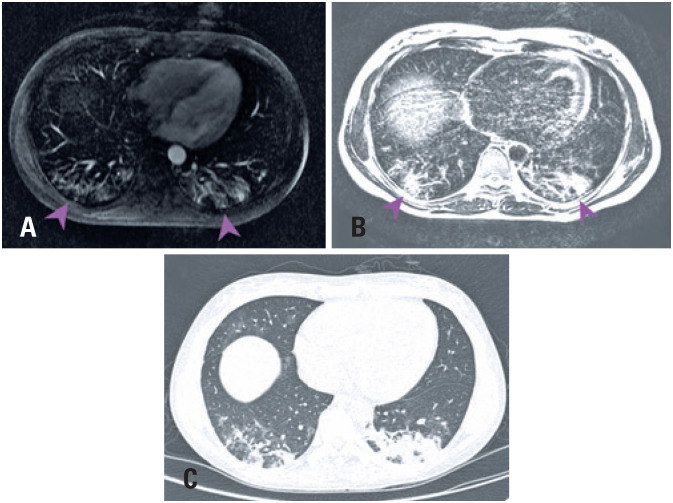
(A) axial upper abdominal computed tomography in T2 sequence ground-opacities showing subpleural and posterior predominance, suspected by COVID-19; (B) changes in the frame of the same resonance T2-weighted image, making findings more evident, similar to the one expected in computed tomography; (C) chest computed tomography of the patient at the same level showing a great similarity featured changes in magnetic resonance cuts

Magnetic resonance imaging cuts (Figure 2) showed bilateral ground-glass opacities in lung bases with posterior and subpleural predominance with similar aspects to those showed in the CT.

A viral infection when presenting lung manifestations often can be similar and indistinguishable pattern among them. In case of COVID-19, we know the typical pattern is peripheral and bilateral ground-glass, or round and multifocal, associated with or not to consolidation and septal lines, often predominant in the posterior aspect of the lower lobes.^(^^1^^)^ The signal of reversed halo can appear more lately in time.

Although chest magnetic resonance (MR) is seldom-used method and often not indicated for lung pulmonary assessment in suspected cases of COVID-19, this method can be adopted to analyze radiological signs in the lung parenchyma that indicate the presence of this disease in patients who conducted a study for other reasons. The radiologist needs to understand these findings and be alert to recognize them in cases with confusing respiratory picture. In our case, we observed ground-glass opacities with intermediate signal in T2 in lung bases, such as the one already reported^(^^2^^)^ in a tomography study with similar aspects and distributions. In cases that similar findings of the present report are detected in patients without diagnostic and who would be conducting MR for other reasons, *i.e.*, to investigate abdomen and dorsal spine, there is a need to inform the physician assistant about patterns in the lungs as markers of COVID-19 infection, especially in times of pandemic. This would help to establish rapid therapeutic approach, immediate isolation of the infected individual, and consequently, reduction in person-to-person transmission and organization of close monitoring.
