# Supplementary material for: COVID-19 on resonance magnetic: an incidental but important finding in times of pandemic
Source: Einstein (Sao Paulo). 2020 Nov 5;18:eAI5891. doi: 10.31744/einstein_journal/2020AI5891 (PMC7647385; doi:10.31744/einstein_journal/2020AI5891)
Supplement: Translation

# Translation: COVID-19 na ressonância magnética: um achado incidental, mas importante em tempos de pandemia

**Authors:** José Vitor Rassi Garcia, Eduardo Kaiser Ururahy Nunes Fonseca, Rodrigo Caruso Chate, Daniel Giunchetti Strabelli, Lucas de Pádua Gomes de Farias, Bruna Melo Coelho Loureiro, Lorena Carneiro Ferreira, Márcio Valente Yamada Sawamura

**Affiliations:** 1 São PauloSP Brasil Hospital das Clínicas, Faculdade de Medicina, Universidade de São Paulo, São Paulo, SP, Brasil.

Paciente do sexo feminino, 34 anos, com antecedente pessoal de retocolite ulcerativa há 5 anos, em uso de sulfassalazina e mesalazina, deu entrada no pronto-socorro apresentando quadro de astenia, dispneia, febre e tosse associada a escarro hemoptoico há 8 dias, saturando 97% ao ar ambiente. Realizou tomografia computadorizada (TC) (Figura 1) que demonstrou opacidade em vidro fosco esparsa, embora mais evidente no contorno posterior dos lobos inferiores. Foi admitida para internação e teve o diagnóstico de infecção pelo novo coronavírus por meio de reação em cadeia da polimerase com transcrição reversa (rt-PCR). Durante a internação, apresentou dor abdominal difusa com fezes líquidas, escurecidas e de odor forte, realizando ressonância magnética (RM) do abdome superior para avaliação.

**Figura 1:** Corte axial da tomografia computadorizada de tórax sem contraste, evidenciando opacidades bilaterais nas bases pulmonares, com predomínio periférico e posterior, suspeitas de acometimento por COVID-19

Os cortes da RM (Figura 2) evidenciaram opacidades bilaterais nas bases pulmonares, com predomínio posterior e subpleural, com aspecto similar àquele demonstrado na TC.

**Figura 2:** (A) corte axial da ressonância magnética de abdome superior em sequência T2 evidencia opacidades bilaterais nas bases pulmonares, com predomínio posterior e subpleural, suspeitas para acometimento por COVID-19; (B) alteração de janela da mesma imagem ponderada em T2 de ressonância, tornando os achados mais conspícuos e evidentes, similares ao esperado em uma tomografia computadorizada; (C) imagens da tomografia computadorizada do tórax da paciente no mesmo nível, demonstrando a grande semelhança das alterações caracterizadas nos cortes de ressonância magnética

As infecções virais, quando apresentam manifestação pulmonar, geralmente podem se mostrar com padrão semelhante e indistinguível entre si. No caso da COVID-19, sabe-se que o padrão típico é o de vidro fosco periférico e bilateral, ou arredondado e multifocal, associado ou não a consolidações e linhas septais de permeio, frequentemente predominando no aspecto posterior dos lobos inferiores.^(^^1^^)^ O sinal do halo invertido pode aparecer mais tardiamente.

Apesar de a RM de tórax ser um método pouco utilizado e não ser indicada para avaliação pulmonar em casos suspeitos de COVID-19, é possível analisar sinais radiológicos no parênquima que indiquem a presença da doença em pacientes que realizam o estudo por outros motivos, devendo o médico radiologista conhecer esses achados e estar atento para seu reconhecimento em casos sem nítido quadro respiratório. No caso demonstrado, vemos as opacidades com sinal intermediário em T2 nas bases pulmonares, tal qual relatado,^(^^2^^)^ já demonstradas no estudo tomográfico e com aspecto e distribuição superponíveis. Em casos em que achados como esse sejam detectados em pacientes sem o diagnóstico e que estejam realizando RM por outros motivos, como, por exemplo, estudos do abdome ou da coluna dorsal, faz-se necessário informar o médico assistente do padrão de imagem suspeita para acometimento pulmonar para a COVID-19, sobretudo em tempos de pandemia, para que uma abordagem terapêutica integrada seja prontamente formulada, e o adequado isolamento do doente seja realizado, minimizando a infecção de terceiros e realizando o acompanhamento próximo.
